# Complete Genome Sequence of *Edwardsiella piscicida* Isolate S11-285 Recovered from Channel Catfish (*Ictalurus punctatus*) in Mississippi, USA

**DOI:** 10.1128/genomeA.01259-16

**Published:** 2016-11-23

**Authors:** Stephen R. Reichley, Geoffrey C. Waldbieser, Hasan C. Tekedar, Mark L. Lawrence, Matt J. Griffin

**Affiliations:** a College of Veterinary Medicine, Mississippi State University, Mississippi, USA; b Aquatic Research and Diagnostic Laboratory, Thad Cochran National Warmwater Aquaculture Center, Stoneville, Mississippi, USA; c USDA-ARS Warmwater Aquaculture Research Unit, Thad Cochran National Warmwater Aquaculture Center, Stoneville, Mississippi, USA

## Abstract

*Edwardsiella piscicida* is a recently described Gram-negative facultative anaerobe and an important pathogen to many wild and cultured fish species worldwide. Here, we report the complete and annotated genome of *E. piscicida* isolate S11-285 recovered from channel catfish (*Ictalurus punctatus*), consisting of a chromosome of 3,923,603 bp and 1 plasmid.

## GENOME ANNOUNCEMENT

The genus *Edwardsiella* was first recognized in the 1960s with the description of *E. tarda* (1, 2). Over the following decades, two additional taxa in the genus were described, *E. hoshinae* and *E. ictaluri* (3, 4). Of the group, *E. tarda* was historically considered the most diverse and widespread species (5, 6). Recent investigations into its heterogeneity revealed that this previous classification actually encompassed three genetically distinct, yet phenotypically ambiguous, taxa, namely, *E. tarda*, *E. piscicida*, and *E. anguillarum* (7, 8). *Edwardsiella piscicida* has since been isolated from a variety of diseased wild and cultured fish (9–12). Recent studies demonstrated that *E. piscicida* is more virulent to channel catfish than *E. tarda* or *E. anguillarum* and is increasingly recovered from diseased farm-raised catfish in the southeastern United States (13, 14). The identity of *E. piscicida* isolate S11-285 was confirmed in previous research using multilocus sequencing, repetitive extragenic palindromic PCR, and species-specific PCR (13, 15).

Genomic DNA was sequenced using Pacific Biosciences (PacBio) technology to 140× coverage. After correction of reads, 25× genome coverage was assembled into four contigs using Canu version 1.0 (16). Illumina sequences (30× coverage, minimum depth of five) were mapped to the PacBio assembly using BWA version 0.7.10-r789 (17); base errors and insertions/deletions were corrected using Pilon version 1.16 (18) iteratively until no further base corrections were made automatically. Ribosomal RNA genes were sequenced using Sanger sequencing of cloned PCR products spanning the rRNA loci; these genes were then aligned to the genomic contigs to produce a single contig. The genome sequence was circularized and relinearized at a position 1 M bases downstream. Illumina and PacBio sequences were realigned and visualized using the Integrated Genome Viewer (19) for validation of contiguity. The S11-285 plasmid was sequenced by the Massachusetts General Hospital Center for Computational and Integrative Biology (MGH CCIB) (https://dnacore.mgh.harvard.edu) and assembled using the MGH CCIB NGS *de novo* assembler UltraCycler version 1.0 (B. Seed and H. Wang, unpublished data).

The complete genome was submitted to the NCBI Prokaryotic Genome Annotation Pipeline (PGAP) for annotation and submission to GenBank. Furthermore, the genome was submitted for Rapid Annotations using Subsystems Technology (RAST) analysis (20, 21) with the Glimmer option. Average nucleotide identities (ANI) (22) and digital DNA-DNA hybridization (dDDH) estimations (23) were determined using online calculators (ANI, http://enve-omics.ce.gatech.edu/ani/; dDDH, http://ggdc.dsmz.de/distcalc2.php).

The *E. piscicida* genome consists of one circular chromosome of 3,923,603 bp (59.6% G+C content) and 1 plasmid of 3,164 bp (48.2% G+C content). PGAP annotation predicted 3,509 genes encoding 3,293 proteins. RAST analysis predicted 497 subsystems with 3,779 coding sequences and 136 RNAs. *E. piscicida* isolate S11-285 shares an ANI of 99.5% (dDDH, 92.8%) with *E. piscicida* isolate ET883 (*E. piscicida* type strain; GenBank accession no. GCF_000804515.1) (7), 94.6% (dDDH, 59.4%) with *E. anguillarum* isolate 080813 (*E. anguillarum* type strain; GenBank accession no. CP006664) (8, 24), 92.2% (dDDH, 48.2%) with *E. ictaluri* isolate 93-146 (GenBank accession no. CP001600) (25), and 83.4% (dDDH, 25.5%) with *E. tarda* isolate FL95-01 (GenBank accession no. CP011359) (26).

### Accession number(s).

The complete genome sequence for *Edwardsiella piscicida* isolate S11-285 has been deposited in GenBank under accession no. CP016044 and its plasmid deposited under accession no. CP016445.
